# Case Report: Simultaneous pancreas–kidney transplantation in MELAS: first reported case with 5-year follow-up

**DOI:** 10.3389/frtra.2026.1737352

**Published:** 2026-01-22

**Authors:** Vera Nilsén, Julia Bojstedt, Johan Nordström

**Affiliations:** 1 Division of Transplantation Surgery, CLINTEC, Karolinska Institutet, Stockholm, Sweden; 2 Department of Transplantation Surgery, Karolinska University Hospital, Stockholm, Sweden; 3 Division of Perioperative Medicine and Intensive Care (PMI), Karolinska University Hospital, Stockholm, Sweden

**Keywords:** graft survival, long-term outcomes, MELAS, mitochondrial disease, mitochondrial disorder, mtDNA-disorder, simultaneous pancreas-kidney transplantation

## Abstract

**Background:**

Mitochondrial encephalomyopathy with lactic acidosis, and stroke-like episodes (MELAS) is a rare mitochondrial DNA disorder that, in severe cases, can result in insulin-dependent diabetes and end-stage renal disease (ESRD). While organ transplantation is a potential treatment, documented cases remain scarce.

**Methods:**

A 40-year-old patient with dialysis-dependent ESRD and diabetes secondary to MELAS underwent simultaneous pancreas-kidney transplantation. The perioperative and postoperative periods were uncomplicated with only targeted MELAS-specific adaptations to standard protocols.

**Results:**

During the 5-year follow-up, the patient maintained excellent kidney allograft function and sustained insulin independence, with no need for dialysis or exogenous insulin therapy. At 5 years, creatinine was 77 µmol/L with an estimated GFR above 90 mL/min/1.73 m^2^, and glycated hemoglobin was 40 mmol/mol.

**Conclusions:**

SPK transplantation may be feasible in carefully selected patients with MELAS, ESRD, and diabetes, providing durable renal and metabolic graft function at 5 years. To our knowledge, this is the first reported SPK case in MELAS, with extended follow-up.

## Introduction

Mitochondrial encephalopathy, with lactic acidosis, and stroke-like episodes (MELAS) is a mitochondrial disorder caused by mutation in the mitochondrial genome, following a maternal inheritance pattern and exhibiting variable progression. The estimated incidence is 1 in 4,000 (1, 2). The most common mutation, m.3243A>G, occurs in the MT-TL1 gene, which encodes mitochondrial tRNA(Leu(UUR)). This dysfunction disrupts the electron transport chain, predominantly affecting high-energy-dependent organs. Consequently, the inability to generate sufficient energy can result in multi-organ failure (1, 3). The primary features of MELAS include stroke-like episodes, seizures, cerebellar ataxia. Additional manifestations include insulin-dependent diabetes mellitus, cardiomyopathy, deafness, pigmentary retinopathy and cerebellar ataxia (2). Current treatment options are limited to supportive and symptomatic care. L-Arginine has been shown to reduce frequency of stroke-like episodes, while coenzyme Q10 and its analogues are suggested to enhance mitochondrial energy production (4, 5). It has been suggested that replacing calcineurin inhibitors with mTOR inhibitors as immunosuppressive therapy could be beneficial for MELAS patients who have undergone kidney transplantation, as it has been shown to improve mitochondrial function (6).

Monitoring disease progression is crucial, as kidney failure among MELAS patients is most attributed to focal segmental glomerulosclerosis (FSGS) or diabetic nephropathy (5). Given the multisystemic and progressive nature of MELAS, transplant candidacy should explicitly address neurological trajectory, metabolic reserve, and the potential mitochondrial toxicity of perioperative and immunosuppressive medications. Simultaneous pancreas-kidney (SPK) transplantation is the preferred treatment option for selected patients with type 1 Diabetes Mellitus (T1DM) and secondary end-stage renal disease (ESRD). It is the only curative treatment for T1DM, offering glucose control without the need for insulin therapy and improved long-term survival (7). In the literature, case reports have documented successful outcomes and improved quality of life in patients with MELAS undergoing single-organ transplantation, such as kidney or heart transplants, for ESRD or cardiomyopathy (8–10). However, to our knowledge, no previous reports of SPK transplantation in a patient with MELAS, with a follow-up period of 5 years, have been published.

We present the case of an SPK transplantation from a standard-criteria brain-dead donor to an adult patient with MELAS syndrome, diabetes mellitus and end-stage renal disease requiring hemodialysis.

## Case description

The recipient was a 40-year-old man diagnosed with MELAS syndrome, confirmed 8 years prior to transplantation through muscle biopsy, which revealed a high mutation load (A3243G, 80%–90% in muscle). The patient had been diagnosed with insulin-dependent diabetes mellitus 10 years before transplantation, and pre-transplant C-peptide levels were within the normal range, consistent with mitochondrial diabetes. According to the American Diabetes Association, mitochondrial diabetes is classified under “Other; genetic defects of the beta cell” and accounts for up to 3% of all diabetes cases (11–14).

The patient also presented with severe diabetic retinopathy, small-fiber neuropathy, and bilateral sensorineural hearing impairment. A kidney biopsy 8 years before the transplant confirmed diabetic nephropathy, and renal function progressively declined, necessitating peritoneal dialysis 6 months before transplantation. Baseline characteristics and pre-transplant investigations are summarized in Table 1. At the time of transplant, the recipient's BMI was 23 kg/m^2^ and he was low immunized, and the patient was considered at low immunologic risk with and a low panel-reactive antibody level and no known donor-specific antibodies.

**Table 1:** Timeline of key clinical events and laboratory parameters before and after simultaneous pancreas–kidney (SPK) transplantation in a man with MELAS.

Timepoint	Event	Creatinine	eGFR	B-Glucose	C-Peptide	Pancreatic amylase	HbA1C
-10 y pre-op	Diabetes diagnosis	75	>90	28	0.54	–	127
-8 y pre-op	MELAS diagnosis	84	>90	5.6	0.44	–	48
-6 m pre-op	Dialysis start	708	10	7.8	–	–	48
-1 day pre-op	Pre SPK	660	10	6.3	1.5	0.18	45
POD 7	Inpatient course	85	87	6.3	4.9	1.71	42
POD 30	Short-term follow-up	96	78	5.9	2.2	0.79	30
POD 90	Short-term follow-up	100	75	5.4	2.0	0.71	33
POD 180	Mid-term follow-up	103	69	4.8	1.6	0.68	39
Post-op 1 year	Mid-term follow-up	97	78	5.2	2.0	0.8	39
Post-op 2 years	Long-term follow-up	97	76	5.1	2.1	0.58	43
Post-op 3 years	Long-term follow-up	91	81	4.8	2.7	0.61	40
Post-op 4 years	Long-term follow-up	91	80	5.4	1.4	0.56	39
Post-op 5 years	Long-term follow-up	77	>90	5.4	2.6	0.5	40

y; year m; months d; day POD; post-operative day.

Reference ranges (males <50 years): Creatinine (60–100 µmol/L) eGFR (>90 mL/min/1.73) B-glucose (4–6 mmol/L) C-peptide (0.26–1.73 nmol/L) Pancreatic Amylase (0.15–1.1 µkat/L) HbA1c (27–42 mmol/mol).

During the pre-transplant evaluation, a living kidney donor was considered, and the patient's sister was evaluated. However, she was excluded as a donor due to the presence of the MELAS mutation, although in a less severe form. After comprehensive anesthetic, cardiovascular, and metabolic assessments, the patient was deemed suitable for SPK. Particular attention was given to managing potential systemic effects of MELAS syndrome during the perioperative period, including the risk of lactic acidosis, gastroenterological disturbances, stroke, and seizures (15). A plan was developed to minimize these risks, including the use of continuous nutritional intravenous infusions during fasting and the avoidance of specific medications such as propofol.

After 4 months on the transplant waiting list, a suitable deceased donor became available, and the patient was admitted for the SPK transplant procedure. The donor was a standard criteria brain dead multi organ donor, a 16-year-old female with a BMI of 25 kg/m^2^ and no relevant medical history. Death occurred after asphyxiation with subsequent anoxic cardiac arrest and hypoxic ischemic brain injury. The HLA mismatch was 3–2 (AB-DR), no donor specific antibodies were detected, and immunological crossmatch tests were negative. CMV serostatus was D-R+ and EBV serostatus was D+R-.

### Surgery

The surgical procedures adhered to well-established protocols (16). No MELAS-related surgical modifications were undertaken, as preoperative assessment identified no anatomical or technical factors requiring deviation from standard SPK surgery. The pancreas preparation included splenectomy and arterial reconstruction using a Y-graft from the donor's iliac arteries, without portal vein extension. A midline incision exposed distal vena cava and right common iliac artery for porto-caval and arterial anastomosis, followed by an entero-entero anastomosis between the duodenal graft and jejunum. Renal transplantation was performed via the midline laparotomy in a standard fashion, with the renal vessels anastomosed to the left common iliac vessels. The ureteral anastomosis was performed to the urine bladder, and the kidney was placed in a retroperitoneal pocket.

## Results/diagnostic assessment

The surgical procedure was completed without complications. Both kidney- and pancreas graft function were rapidly restored and have remained excellent. The patient received immunosuppression according to protocol including Thymoglobulin induction (total 5 mg/kg body weight, divided into two doses administered perioperatively and on postoperative day 2), followed by maintenance immunosuppression with tacrolimus, mycophenolate mofetil and corticosteroids. Tacrolimus through targets were 8–12 ng/mL during the first 3 months and 5–8 ng/mL thereafter, with regular through-level monitoring. Infection prophylaxis included trimethoprim-sulfamethoxazole for 6 months for Pneumocystis jirovecii pneumonia, valganciclovir for 3 months for CMV and nystatin for 1 month.

Anticoagulation was managed with acetylsalicylic acid and low molecular weight heparin. In this case, no consideration of potiential drug interactions between immunosuppressive medications and mitochondrial therapies was required, since the patient was not receiving any mitochondrial specific pharmacotherapy, during the perioperative period or throughout the follow-up.

Although due to the underlying mitochondrial disorder, the peri- and postoperative course were anticipated to be complicated, particularly concerning risks of cerebral events and lactate accumulation. To mitigate these risks, postoperative fasting episodes were to be strictly avoided. Nevertheless, a four-hour fast on postoperative day 1 for C-peptide measurement resulted in a transient rise in serum lactate from 0,9 to 2,4 mmol/L. No adverse symptoms were observed, and lactate levels normalized after resuming parenteral nutrition. The following postoperative course was uneventful, with creatinine levels normalizing, no need for insulin, and the absence of early surgical complications. Standard postoperative monitoring, as applied to all SPK recipients at our center, was performed to enable early detection of clinical changes through regular assessment of laboratory test, vital signs and immunosuppressive drug concentrations. The patient was discharged on post-operative day 11. Early post-operative laboratory results and subsequent follow-up measurements are shown in Table 1.

Throughout the 5-year follow-up, serum creatinine and C-peptide levels remained stable, and the patient required neither dialysis nor exogenous insulin, and no rejection episodes occurred. The patient´s quality of life improved, and despite an extended sick leave related to the COVID-19 pandemic, he returned to work. Neurological assessments indicated continued tolerance to tacrolimus without evidence of MELAS symptom progression.

This case represents, to our knowledge, the first reported successful SPK in a patient with diabetes mellitus and end-stage renal disease secondary to the mitochondrial disorder MELAS.

## Patient perspective

The patient reported that becoming dialysis-independent and no longer requiring insulin therapy was life-changing. He expressed gratitude that the transplantation was successful, with improved quality of life and stable neurological symptoms without progression. He also emphasized that he has been able to return to work and live a good life.

## Discussion

Organ transplantation remains the definitive treatment for end-stage organ failure. In patients with type 1 diabetes mellitus and secondary end-stage renal disease, simultaneous pancreas kidney transplantation is the treatment of choice and offers durable insulin independence and improved long-term outcomes. In MELAS, however, diabetes typically reflects a genetic defect in beta-cell function rather than autoimmune type 1 diabetes as classified by the American Diabetes Association. Consequently, applying SPK transplantation to a progressive multisystem mitochondrial disorder raises important concerns regarding perioperative metabolic vulnerability, neurological prognosis, and potential medication related mitochondrial toxicity.

Before listing the patient for transplantation, we performed an extensive literature review. Although successful kidney and heart transplantation in MELAS have been reported (8–10), outcomes after SPK transplantation have not been documented. Perioperative and anesthetic challenges in MELAS have been highlighted previously (17), underscoring the need for proactive risk mitigation.

As shown in Table 1, kidney function recovered rapidly and glycemic control remained stable after transplantation. At five years, both kidney and pancreas graft function remain excellent, with continued dialysis independence and no requirement for exogenous insulin. Importantly, neurological symptoms have remained stable postoperatively, and the patient has returned to work.

Our experience suggests that SPK transplantation can be feasible in carefully selected MELAS patients when MELAS-specific perioperative precautions are implemented. In this case, risk reduction focused on strict avoidance of prolonged fasting, early nutritional support, and close monitoring of lactate and acid–base balance, as even brief fasting may provoke metabolic stress. In addition, anesthetic planning emphasized avoidance of physiologic triggers that may precipitate neurological events, including hypoglycemia, hypotension, hypoxia, and infection.

While limited evidence suggests that mTOR inhibitors may be beneficial in mitochondrial disease and could potentially influence neurological progression in some transplant recipients (6), we have not modified the current regimen given the absence of neurological deterioration and the excellent graft outcomes. In the absence of rejection episodes, the immunosuppressive strategy has therefore been maintained.

## Conclusion

Patients with mitochondrial disorders such as MELAS, who develop secondary Diabetes Mellitus and end-stage renal disease, may be suitable candidates for SPK, deriving substantial benefits from the procedure. Special considerations in perioperative care are essential, given the metabolic complexities in this patient population.

## Data Availability

The original contributions presented in the study are included in the article/Supplementary Material, further inquiries can be directed to the corresponding author.
